# Vascular Complications in Patients with Chronic Pancreatitis

**DOI:** 10.3390/jcm10163720

**Published:** 2021-08-21

**Authors:** Miroslav Vujasinovic, Ana Dugic, Amar Nouri, Torkel B Brismar, Francisco Baldaque-Silva, Ebba Asplund, Wiktor Rutkowski, Poya Ghorbani, Ernesto Sparrelid, Hannes Hagström, J.-Matthias Löhr

**Affiliations:** 1 Department of Upper Abdominal Diseases, Karolinska University Hospital, 141 86 Stockholm, Sweden; fbaldaquesilva@gmail.com (F.B.-S.); wiktor.rutkowski@ki.se (W.R.); poya.ghorbani@sll.se (P.G.); ernesto.sparrelid@ki.se (E.S.); hannes.hagstrom@ki.se (H.H.); matthias.lohr@ki.se (J.-M.L.); 2 Department of Medicine, Huddinge, Karolinska Institutet,141 86 Stockholm, Sweden; ana.dugic@ki.se (A.D.); amar.nouri@ki.se (A.N.); ebba.asplund@ki.se (E.A.); 3 Department of Radiology, Karolinska University Hospital, 141 86 Stockholm, Sweden; torkel.brismar@gmail.com; 4 Department of Clinical Science, Intervention, and Technology (CLINTEC), Karolinska Institutet, 141 86 Stockholm, Sweden; 5 Clinical Epidemiology Unit, Department of Medicine, Solna, Karolinska Institutet, 171 77 Stockholm, Sweden

**Keywords:** chronic pancreatitis, splanchnic circulation, hepatic vein thrombosis, pseudoaneurysm, vascular complications

## Abstract

Introduction: Chronic pancreatitis (CP) is a long-standing progressive inflammation of the pancreas, which can lead to a variety of vascular complications, such as splanchnic venous thrombosis (VT) and arterial pseudoaneurysm (PA). There is a lack of studies on vascular complications in Scandinavian countries. Methods: We performed a retrospective analysis of medical records of patients with CP identified from the Karolinska University Hospital database between 2003 and 2018. A total of 394 patients with definite CP were included in the study. Results: There were 33 patients with vascular complications, with a median age of 62 (IQR 55–72) years. The cumulative incidence of vascular events was 3.2% at 5 years. Thirty patients had isolated VT, whereas three patients had PA (7.6% and 0.8%, respectively). Isolated splenic vein thrombosis was most common (53.3%), followed by a combination with other splanchnic veins. PA was found in the splenic artery in two patients and in the left gastric artery in one patient. Varices were present in three (10%) patients; variceal bleeding was not recorded. All patients had asymptomatic splanchnic VT, most with chronic VT with developed collaterals (83.3% had abdominal collateral vessels). Nearly two-thirds of patients with VT (63.3%) received no treatment, whereas 11 (36.6%) were treated with anticoagulants. Pseudocysts and alcoholic etiology of CP are risk factors for vascular complications. Conclusions: The cumulative incidence of vascular complications was 3.2% at 5 years. Splanchnic VT is more common than PA. Patients were asymptomatic with no variceal bleeding, explained by well-developed collateral vessels and strong study inclusion criteria.

## 1. Introduction

Chronic pancreatitis (CP) is characterized by the progressive inflammation of the pancreas, which can lead to a variety of life-threatening long-term complications [1]. Vascular sequelae of CP include splanchnic venous thrombosis (VT) and arterial pseudoaneurysm (PA).

Consequences of pancreatitis-associated splanchnic VT are manifested in a pathophysiological pathway starting with a localized form of portal hypertension, venous hypertension in the splenoportal and/or gastroepiploic systems ultimately leading to oesophageal, gastric or colonic varices [2]. A systematic review and meta-analysis of observational studies showed a pooled prevalence of splanchnic VT of 13.6% in all types of pancreatitis, with a pooled prevalence of splanchnic VT of 16.6% and 11.6% in patients with acute and chronic pancreatitis, respectively [2]. Risk factors for splanchnic VT include persistent acquired risk factors (liver cirrhosis, cancers, inflammatory bowel disease, antiphospholipid syndrome, autoimmune diseases), transient acquired risk factors (abdominal surgery, hormonal therapy, pregnancy, puerperium, abdominal infections) and inherited risk factors (factor V Leiden mutation, protein C deficiency, protein S deficiency, antithrombin deficiency, JAK2V617F mutation, prothrombin G20210A mutation) [3]. The prevalence of splanchnic VT in acute and chronic pancreatitis was higher (16.9%) in Europe, compared to studies from America (11.5%) and Asia (8.5%) [2].

There is significant variability in the reported risk for arterial PA in pancreatitis. A recently published meta-analysis showed the pooled incidence rates of PA in acute and chronic pancreatitis were 0.05% and 0.03%, respectively [4]. Angiographic embolization is the method of choice for the treatment of PA [1] and shows a high technical and clinical success rate [4].

There is a lack of studies on vascular complications in Scandinavian countries. We aim to fill the knowledge gap on VT and PA through a retrospective analysis of patients with CP in a high-volume tertiary center.

## 2. Methods

### 2.1. Patients

We performed a retrospective analysis of medical records of patients presented with CP at the Department of Upper Abdominal Diseases at Karolinska University Hospital. Patients with ICD codes for CP (K86.0 and K86.1) were identified in our database between 2003 and 2018. The definite diagnosis of CP was determined according to M-ANNHEIM criteria [5].

**Figure 1:**
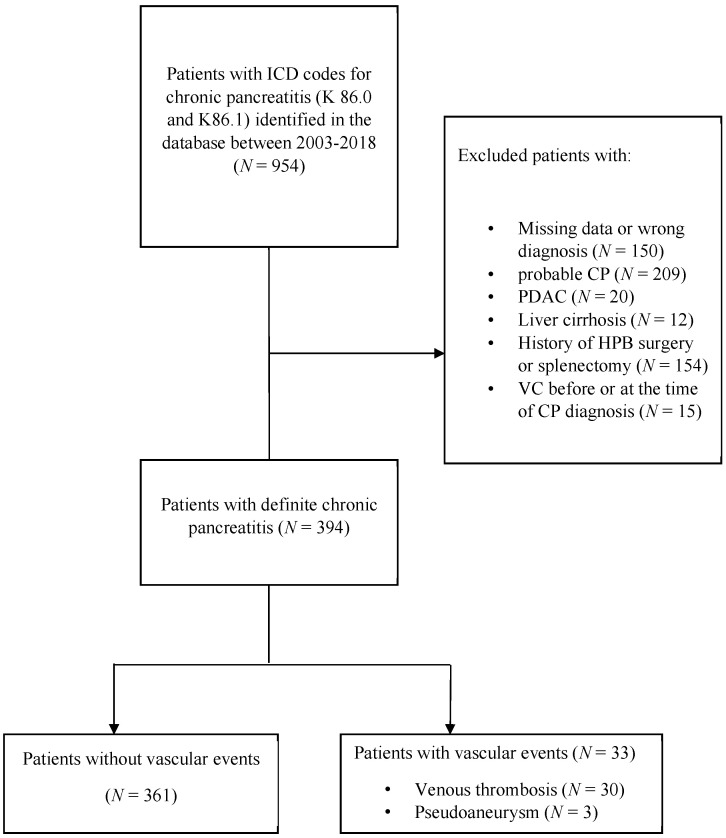
Flow chart of patient selection. CP—chronic pancreatitis; PDAC—pancreatic ductal adenocarcinoma; HPB—hepato-pancreatico-biliary.

We excluded patients <18 years at the time of data analysis, patients with cancers in the abdominal cavity, patients with cirrhosis of the liver, patients without a Swedish personal identity number, patients with probable CP according to M-ANNHEIM criteria, patients diagnosed with vascular complications before or at the time of CP diagnosis, and patients who have previously undergone pancreatic surgery or splenectomy. We initially identified 954 patients with CP, and after application of the above-mentioned exclusion criteria, 394 patients were included in the final analysis (Figure 1).

### 2.2. Definitions

Definite CP was diagnosed by imaging (computed tomography (CT), magnetic resonance imaging (MRI) or both, with one or more of the following criteria: (a) pancreatic calcifications, (b) moderate or marked ductal lesions, (c) marked and persistent exocrine insufficiency defined as pancreatic steatorrhea markedly reduced by enzyme supplementation or (d) typical histology of an adequate histological specimen). 

Vascular complications (VT and PA) were detected by contrast enhanced CT, MRI or both. We used the data from institutional imaging reports performed and signed by two independent radiologists according to routines at our hospital.

### 2.3. Follow Up

Follow-up began on the date of CP diagnosis. Data were censored at the time of the last clinical contact with the patient or death.

### 2.4. Antithrombotic Treatment

Patients were treated with anticoagulant therapy according to local clinical practice. The standard initial treatment was weight-adapted subcutaneous application (200 IE/kg/day) of low-molecular-weight heparin (LMWH) until occurrence of collateral vessels. The duration of therapy was determined by the treating physician during follow-up visits. The patients who had developed abdominal collaterals prior to detection of thrombosis were considered to have an old thrombosis and did not receive treatment.

### 2.5. Statistics

Categorical data were presented as total numbers and proportions, whereas continuous data were reported as median with interquartile range. After the cohort was stratified based on the occurrence of vascular events, bivariate comparisons at baseline were performed. Categorical variables were tested by chi-square test or Fisher’s exact test, as appropriate. The Mann–Whitney U test was used for continuous variables, after normality was assessed by Shapiro–Wilk test.

The Multivariable Cox proportional hazard model was used to determine risk factors for vascular events in patients with CP. Demographic and clinical characteristics that were clinically and statistically significant (alcohol consumption, history of smoking, acute pancreatitis prior to CP and presence of pseudocyst) were assessed as risk factors by univariate analysis. Variables significant at the univariate analysis were included in the final (multivariable) model. Comparisons were presented as hazard ratios (HRs) and 95% confidence intervals (CIs). 

The Kaplan–Meier method was employed to calculate the cumulative incidence of vascular events at 5, 10, and 15 years from CP diagnosis. The analyses were performed using the software IBM SPSS Statistics, Version 27.0. The *p*-values < 0.05 (two-sided) were considered statistically significant.

### 2.6. Ethics

The study was approved by the Regional Ethics Committee in Stockholm, registration number 2020-02209. The requirement for individual informed patient consent was waived by the committee owing to the retrospective nature of the study.

## 3. Results

**Figure 2:**
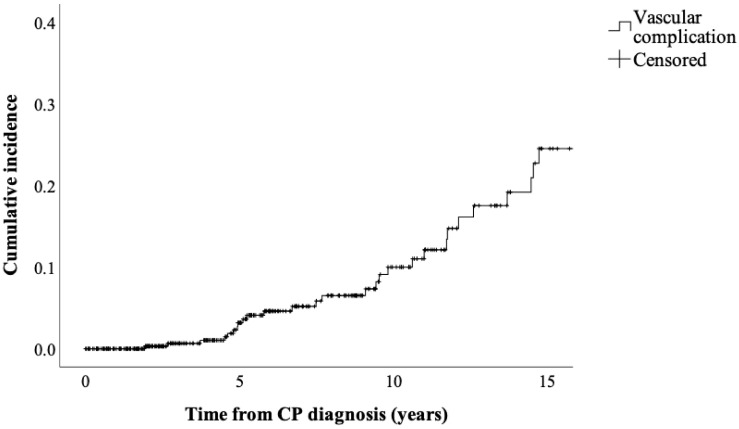
Cumulative incidence of vascular complications in patients with CP. Cumulative incidence was 3.2% at 5 years, 10% at 10 years and 24.5% at 15 years. CP—chronic pancreatitis.

The study included a total of 394 patients with definite CP (Figure 1), with a median follow up of 5.4 (IQR 2.5–9.9) years. The cumulative incidence of vascular complications was 3.2% at 5 years, 10% at 10 years and 24.5% at 15 years (Figure 2). There were 33 patients who experienced vascular events (Table 1), with a median age 62 (IQR 55–72) years. Thirty patients had isolated VT, whereas three patients had PA alone (7.6% and 0.8%, respectively). The most common form of VT was isolated splenic vein thrombosis, followed by the combination with superior mesenteric and portal vein thrombosis (53.3%, 13.3% and 10%, respectively). In two patients, PA was found in the splenic artery and in one patient in the left gastric artery. Varices were present in three (10%) patients; no variceal bleeding was recorded. All of our patients had asymptomatic splanchnic VT, most with chronic VT with developed collaterals (83.3% of patients had abdominal collateral vessels). Nearly two-thirds of patients with VT (63.3%) received no treatment, whereas 11 (36.6%) were treated with anticoagulants (low-molecular-weight heparin (LMWH) or novel anticoagulants (NOAC)). All three patients who presented with varices were not treated with anticoagulants. Among all patients who developed collaterals, no significant difference was noted regarding anticoagulant treatment: 10 (40%) patients received anticoagulants, whereas 15 (60%) patients received no treatment (*p* = 0.622). 

After the cohort was stratified by the presence of vascular complications, significant differences were noted regarding age at CP diagnosis, etiology of CP, smoking, history of acute pancreatitis and occurrence of pseudocyst (Table 2). These variables were identified as risk factors for vascular complications on a univariate analysis. However, after performing a multivariable analysis, only pseudocyst (HR 8.66, CI 3.33–22.54, *p* < 0.001) and alcohol consumption (HR 3.56, CI 1.40–9.03, *p* = 0.007) remained associated with an increased risk of vascular events (Table 3).

## 4. Discussion

Vascular complications are serious consequences of CP that require a careful patient evaluation, diagnostic work-up, assessment of the risk and benefits of treatment and optimal treatment intensity [3]. The cumulative incidence of vascular complications in our CP patients was 3.2% at 5 years, with an increasing trend depending on the disease duration (10% at 10 years and 24.5% at 15 years from CP diagnosis). Thirty patients had isolated splanchnic VT and three patients had PA alone (7.6% and 0.8%, respectively).

A systematic review and meta-analysis that included all studies conducted from 1958 to 2014 on 10,560 patients, showed the prevalence of splanchnic VT in patients with chronic pancreatitis ranging from 3% to 41.7% with a pooled prevalence of 11.6%, and a higher prevalence in Europe (16.9%) [2]. However, the significant heterogeneity of data from various studies and different results between Western and Asian countries, including differences in the population lifestyle and diagnostic level, may bias the final results [2]. Splanchnic VT is a cause of a localized form of portal hypertension (commonly referred to as “sinistral”, “left sided” or “linear”) with the development of a collateral blood flow through the splenoportal or gastroepiploic system resulting in gastric, esophageal or colonic varices [6,7]. We detected varices in 10 % of patients, which was lower compared to the systematic review and meta-analysis [6] that reported 53% of patients found to have varices, 77.3% of which were gastric. In our cohort, all patients were found to have varices in gastric fundus (two out of three patients had both fundus and esophageal varices). However, we had to consider a low number of patients in this sub-group since only three patients were diagnosed with varices. Surprisingly, neither of our patients with splanchnic VT and varices had gastrointestinal bleeding; that is in big contrast with the systematic review and meta-analysis showing the aggregate rate of associated gastrointestinal bleeding been 12.3% [6]. However, a low rate of bleeding was reported in a study from the USA, whose experience suggested that gastric variceal bleeding in pancreatitis-induced splenic vein thrombosis was uncommon (4%) [8]. Even a recently published study from India showed a low occurrence of gastrointestinal bleeding (3.5%), suggesting the presence of collaterals as a reducing factor for bleeding [9]. That is in line with our results since 83.3% of patients developed abdominal collateral vessels, which could explain the absence of variceal bleeding and a low necessity of endoscopic interventions in our cohort during the follow-up (median follow up time since the occurrence of vascular event was 4.2 years). Another explanation for this should be the strong inclusion criteria since we included only patients with definite CP and excluded patients with AP without CP, in contrast with other studies in which patients with AP and CP were included in the analysis. Another discrepancy in our study was related to splenomegaly that was detected in only 20% of our patients compared to 51.9% in the systematic review and meta-analysis [6], and 42–54% in studies that were focused of splenomegaly in splanchnic VT [6,10,11]. These troubling inconsistencies in accurately identifying the hallmark sequelae of splanchnic VT can be at least partially explained by the heterogeneity across studies of the techniques used to identify varices (imaging vs. endoscopy) [6]—we diagnosed varices in all patients with an endoscopy. Another explanation is the heterogeneity in types of pancreatitis included in the studies, mixing the patients of acute pancreatitis (AP) and CP and a lack of data on the severity of AP, recurrent AP and CP (heterogeneity in inclusion criteria). Considering the natural course of patients with AP, showing that 10% of patients with a first episode of AP and 36% of patients with recurrent AP develop CP [12], a clear differentiation of pancreatitis subtypes is necessary to elucidate the discrepancies between the studies on the sequelae of splanchnic VT. Furthermore, it is possible that older studies overestimate the frequency of variceal bleeding due to a limited availability of diagnostic tools [13]. Alcohol consumption and pseudocysts were risks factors for vascular events in our study, confirming previous reports [8,9,13]. The association between pseudocysts and splanchnic VT may be explained by the compression of the splenic vein as well as ongoing local inflammation [13]. The treatment of splanchnic VT aims to achieve vessel recanalization and avoid complications; therefore, the anticoagulation treatment is strongly recommended in all patients with symptomatic VT without absolute contraindications. However, there is still controversy regarding the type, dose and duration of anticoagulant treatment in patients with pancreatitis-induced SVT. Current literature offers heterogeneous level of evidence, mainly owing to the disease rarity, nonspecific clinical presentation and use of different imaging techniques [3]. Patients who have a high risk of bleeding represent a special challenge in terms of treatment. On the other hand, there are substantial inconsistencies in anticoagulant therapy recommendations for patients with asymptomatic or chronic SVT at the time of presentation. Although the majority of these patients usually receive some form of anticoagulation, the individualized treatment approach remains common clinical practice.

Anticoagulants (LMWH or NOAC) were prescribed in 36.6% of our patients, whereas nearly two-thirds of patients (63.3%) received no treatment (all of our patients had asymptomatic splanchnic VT with an imaging appearance of chronic VT with developed collaterals). However, the role of splenectomy in patients with CP and splanchnic VT could not be evaluated in the present study due to the exclusion of patients who had previously undergone hepato-pancreato-biliary surgery or splenectomy. Interestingly, no significant difference was noted regarding the presence of abdominal collaterals between patients who underwent anticoagulant therapy and those who received no treatment (40% vs. 60%, respectively). Although all three patients who developed varices did not receive anticoagulants, we cannot draw conclusions regarding the role of anticoagulants in the prevention of gastrointestinal bleeding due to such a low number of events.

PA occurred in 0.8% of patients. A recently published systematic review and meta-analysis on the efficacy of the endovascular embolization of PA showed a pooled incidence rate of PA in CP of 0.03% with the most common site of PA being the splenic artery (37.7%). In our study, PA exhibited a predilection for the splenic artery (two patients), followed by the left gastric artery (one patient). All patients with PA remained asymptomatic and were incidentally diagnosed on imaging during the follow up.

The retrospective nature of the analysis was the major limitation of the study, as well as missing data on inflammatory markers and the lack of additional etiological evaluation of underlying hypercoagulable conditions (primarily the lack of data on genetic mutations accountable for thrombophilia). The potential effect of hormonal therapy on SVT was not assessed within our cohort, which could represent another drawback. However, our study included one of the largest series of CP patients, using strong inclusion criteria with only definite cases of CP of various etiologies. Reporting on clinical significance, treatment and gastrointestinal bleeding represent the strengths of the study.

## 5. Conclusions

The cumulative incidence of vascular complications in CP patients was 3.2% at 5 years, showing an increasing trend depending on the disease duration. Splanchnic VT was more common than PA. Pseudocysts and alcoholic etiology of CP were risk factors for vascular complications. Variceal bleeding was not recorded, probably due to a large proportion of patients in whom abdominal collateral vessels developed and a stronger inclusion criteria in our study compared to others.

## Figures and Tables

**Table 1:** Distribution, clinical manifestations and treatment modalities of vascular events (venous thrombosis or pseudoaneurysm).

PARAMETERS	*N* (%)
**Total number of vascular events**	33/394 (8.4)
	
**Total VT**	30/394 (7.6)
**Vessel involved**	
Isolated PVT	2/30 (6.7)
Isolated SVT	16/30 (53.3)
Isolated MVT	1/30 (3.3)
PVT and SVT	3/30 (10.0)
PVT and MVT	2/30 (6.7)
MVT and SVT	4/30 (13.3)
SVT, PVT and MVT	2/30 (6.7)
	
**Varices on endoscopy**	3/30 (10.0)
**Variceal location**	
Esophagus and fundus	2/3 (66.7)
Fundus and other sites	1/3 (33.3)
**Variceal treatment**	
Variceal acute endoscopic treatment	0/3 (0.0)
Variceal bleeding-prophylaxis treatment (NSBB)	1/3 (33.3)
	
Splenomegaly	6/30 (20.0)
Abdominal collateral vessels	25/30 (83.3)
	
**Venous thrombosis treatment**	
No treatment	19/30 (63.3)
LMWH	10/30 (33.3)
NOAK	1/30 (3.3)
	
**Clinical manifestation of VT**	
Incidental finding	30/30 (100.0)
Gastrointestinal bleeding	0/30 (0.0)
Intraabdominal bleeding	0/30 (0.0)
	
**Total Pseudoaneurysm**	3/394 (0.8)
**Vessel involved**	
Splenic artery	2/3 (66.7)
Left gastric artery	1/3 (33.3)
	
**Clinical manifestation of pseudoaneurysm**	
Incidental finding	3/3 (100.0)
Intraabdominal bleeding	0/3 (0.0)

VT—venous thrombosis; PVT—portal vein thrombosis; SVT—splenic vein thrombosis; MVT—mesenteric vein thrombosis; LMWH—low-molecular-weight heparin; NOAC—novel oral anticoagulants; NSBB—non-selective beta-blockers.

**Table 2:** Demographic and clinical baseline characteristics of patients with chronic pancreatitis categorized by presence and type of vascular event.

Characteristics	No Complications, *n* = 361/394 (91.6)	Vascular Complications, *n* = 33/394 (8.4)	*p*-Value
Age at time of CP diagnosis, median (IQR)	57.5 (44–69)	52 (43.5–54.5)	0.019 *
Age at the time of occurrence of vascular complication, median (IQR)	/	62.2 (55.1–72)	/
Sex male, *n* (%)	223/361 (61.8)	24/33 (72.7)	0.213
BMI at diagnosis, kg/m^2^, *n* (%)			0.218
≤ 25	157/215 (73.0)	14/23 (60.9)	
>25	58/215 (27.0)	9/23 (39.1)	
Etiology			
Alcohol, *n* (%)	145/361 (40.2)	27/33 (81.8)	<0.001 *
Efferent duct, *n* (%)	40/361 (11.1)	1/33 (3.0)	0.231
Family history of pancreatic disease, *n* (%)	32/294 (10.9)	1/24 (4.2)	0.489
Smoking (active or former), *n* (%)	210/346 (60.7)	27/33 (81.8)	0.017 *
Diabetes at diagnosis, *n* (%)	91/347 (26.2)	6/33 (18.2)	0.311
PEI at diagnosis, *n* (%)	144/361 (39.9)	12/33 (36.4)	0.921
Calcification at diagnosis, *n* (%)	206/342 (60.2)	19/29 (65.5)	0.576
AP before CP diagnosis, *n* (%)	228/359 (63.5)	26/32 (81.3)	0.044 *
Pseudocyst, *n* (%)	97/361 (26.9)	27/33 (81.8)	<0.001 *
Pseudocyst size, *n* (%)			0.435
<5cm	23/44 (52.3)	14/24 (58.3)	
5–10 cm	17/44 (38.6)	6/24 (25.0)	
≥10 cm	4/44 (9.1)	4/24 (16.7)	

BMI—body mass index; PEI—pancreatic exocrine insufficiency; CP—chronic pancreatitis; IQR—inter quartile range; * statistically significant *p*-value.

**Table 3:** Univariate and multivariable analyses for vascular complications during follow-up period among patients with chronic pancreatitis.

Variable	Univariate Analysis		Multivariable Analysis	
	HR (95% CIs)	*p*-Value	HR (95% CIs)	*p*-Value
Alcohol	5.07 (2.09–12.30)	<0.001 *	3.56 (1.40–9.03)	0.007 *
Smoking	3.12 (1.29–7.56)	0.012 *	1.25 (0.49–3.17)	0.643
Prior AP episode	1.11 (0.45–2.77)	0.812	/	/
Pseudocyst	9.58 (3.90–23.53)	<0.001 *	8.66 (3.33–22.54)	<0.001 *

AP—acute pancreatitis; HR—hazard ratio; CI—confidence interval; * statistically significant *p*-value.

## Abbreviations

AP	acute pancreatitis
BMI	body mass index
CI	confidence interval
CP	chronic pancreatitis
IQR	interquartile range
LMWH	low-molecular-weight heparin
MVT	mesenteric vein thrombosis
NOAC	novel oral anticoagulants
NSBB	nonselective beta-blockers
HR	hazard ratio
PA	pseudoaneurysm
PEI	pancreatic exocrine insufficiency
PVT	portal vein thrombosis
SVT	splanchnic vein thrombosis
VT	venous thrombosis
